# Supplementary material for: Xylose Isomerase Depletion Enhances Virulence of Xanthomonas citri subsp. citri in Citrus aurantifolia
Source: Int J Mol Sci. 2023 Jul 15;24(14):11491. doi: 10.3390/ijms241411491 (PMC10380989; doi:10.3390/ijms241411491)
Supplement: Supplementary file 1 [file ijms-24-11491-s001.zip › ijms-2352779-supplementary.pdf]

Supplementary Material

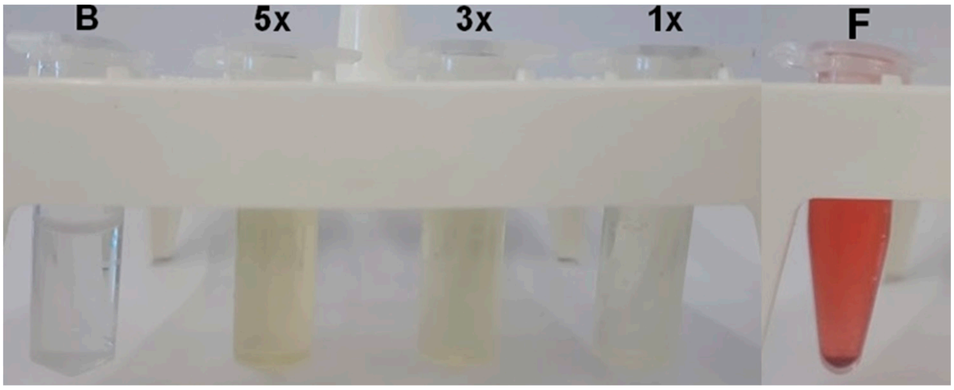

Figure S1. Conversion activity of D-xylose to D-xylulose catalyzed by rXylA2. B | Blank containing buffer and Seliwanoff's reagent. Reactions containing 0.1 M of D-xylose, Seliwanoff's reagent and rXylA2 at concentrations of: **5x** | 0.25 mg.mL^{-1}, **3x** | 0.15 mg.mL^{-1}, and **1x**| 0.05 mg.mL^{-1}; **F** | positive control, composed of 0.1 M of D-fructose, buffer and Seliwanoff's reagent. All buffers used were composed of 50 mM Tris-HCl (pH 8.0), 100 mM NaCl.

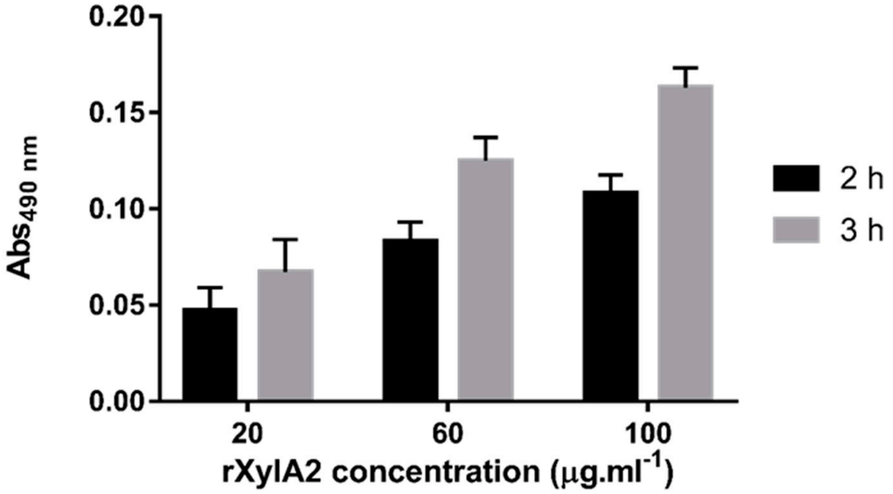

Figure S2. Conversion activity of D-fructose to D-glucose catalyzed by rXylA2. Three assays with different concentrations of the enzyme were performed. The enzyme concentrations used (in µg.ml^{-1}) were 20, 60 and 100. The graph shows the means of measurements performed in triplicate, at 25ºC, after two (black) and three (gray) hours of reaction. Error bars indicate the standard error of the mean (SEM).
